# The Advantages of Lateral Tarsal Strip Procedure

**DOI:** 10.4103/2006-8808.73615

**Published:** 2010

**Authors:** Michele Altieri

When senile involutional entropion develops, some changes are present in the anatomy of the lower lid.[1,2] These changes (mainly worsening of horizontal and vertical lid laxity) cause an imbalance between the usual forces acting on the lower eyelid.[3]

Generally in surgery, an ideal operation should be effective, cause minimal discomfort and morbidity, give an aesthetic result, and have a lasting effect.[4] Lateral strip procedure (LSP) has those characteristics and it does restore normal lid function and give a rapid rehabilitation with few complications and excellent cosmetic outcome.[5,6]

LSP produces a horizontal lid shortening with a diagonal tightening of the orbital septum and lower lid retractors,[7] producing an effective and long lasting improvement of both horizontal and vertical lid laxity.

Olver describes the LSP as an operation in which the entropion repair is performed through only one 1-cm lateral canthal incision. Everting sutures are often performed in combination with LSP for better correction of the inward rotation of eyelid margin.[1,5,7]

Patients affected by entropion and treated with LSP recover very quickly, the cutaneous stitches being removed, in general, five days after the surgery. The postoperative treatment is mild and quick (topical and oral antibiotics for preventing postoperative infections associated with oral paracetamol and/or diclofenac for minimizing the postoperative inflammation of the operated area).

The data and the findings of this paper are presented in a very effective way and confirm, as it is already described in the most important studies about this topic, that this surgical procedure should be commonly performed for the lower lid involutive entropion, also by the general ophthalmologist with a general experience in ophthalmic surgery and not only by the subspecialist ophthalmic plastic surgeon.
